# Post-IVIG Aseptic Meningitis in a Pediatric Patient With Kawasaki Disease: A Case Report

**DOI:** 10.7759/cureus.115091

**Published:** 2026-08-24

**Authors:** Sanae Kheir, Aziza Elouali, Anane Sara, Maria Rkain, Abdeladim Babakhouya

**Affiliations:** 1 Department of Pediatrics, Mohammed VI University Hospital, Oujda, MAR; 2 Faculty of Medicine and Pharmacy, Mohammed First University, Oujda, MAR

**Keywords:** adverse drug reaction, aseptic meningitis, intravenous immunoglobulin (ivig), kawasaki disease (kd), pediatric neurology

## Abstract

Intravenous immunoglobulin (IVIG)-induced aseptic meningitis is a rare but well-recognized neurological complication that may closely mimic bacterial meningitis, making diagnosis challenging. Prompt recognition is essential to avoid unnecessary antimicrobial therapy and prolonged hospitalization.

We report the case of a previously healthy 12-year-old boy who developed aseptic meningitis seven days after receiving high-dose IVIG for Kawasaki disease (KD). He presented with severe headache, vomiting, fever, and mild neck stiffness. Cerebrospinal fluid (CSF) analysis demonstrated neutrophilic pleocytosis (248 cells/mm³, 80% neutrophils) with negative Gram stain and bacterial cultures. Following the exclusion of infectious causes, the patient recovered completely with supportive care alone.

This case highlights that IVIG-induced aseptic meningitis may occur later than the commonly reported 24-72 hours after infusion. Awareness of this uncommon but benign adverse event, together with careful interpretation of CSF findings and exclusion of infectious meningitis, is essential to ensure appropriate management, avoid unnecessary investigations and antibiotic therapy, and reassure clinicians and families regarding its excellent prognosis.

## Introduction

Kawasaki disease (KD) is an acute systemic vasculitis and the leading cause of acquired heart disease in children in developed countries. Early treatment with high-dose intravenous immunoglobulin (IVIG) (2 g/kg) combined with aspirin significantly reduces the risk of coronary artery complications and remains the standard of care [1,2].

Though generally safe and well tolerated, IVIG can cause adverse effects that span from mild infusion-related reactions to rare neurological complications such as aseptic meningitis [3-5]. Aseptic meningitis caused by IVIG is a rare but recognized complication, presenting with signs and symptoms suggestive of bacterial meningitis, such as severe headache, fever, vomiting, and neck stiffness [6]. Examination of cerebrospinal fluid (CSF) typically shows pleocytosis with negative microbiological cultures, and diagnosis is difficult [6].

Early recognition of this benign, self-limiting condition is important to avoid unnecessary workup, prolonged courses of antibiotics, and hospitalization [7,8]. This case is documented to raise awareness of delayed-onset IVIG-induced aseptic meningitis and to emphasize that an atypical temporal presentation should not exclude this diagnosis when the clinical and CSF findings are compatible.

## Case presentation

We report a case of a previously healthy 12-year-old boy, the only child of second-degree consanguineous parents, who was admitted to our pediatric department with KD in April 2026. He had no significant past medical history. He presented with persistent high-grade fever lasting six days, bilateral non-purulent conjunctivitis, diffuse maculopapular rash, and oropharyngeal changes, fulfilling the diagnostic criteria for KD. Echocardiography revealed no coronary artery abnormalities. He received a single infusion of IVIG (2 g/kg) administered over 10 hours, together with aspirin at an anti-inflammatory dose (50 mg/kg/day) for five days, followed by antiplatelet-dose aspirin. Oral prednisone (1 mg/kg/day) was administered as adjunctive corticosteroid therapy for five days in order to improve control of the systemic inflammatory response and lower the risk of disease-related consequences. This treatment resulted in rapid clinical improvement, and the patient was discharged after six days of hospitalization. Inflammatory markers progressively improved, with C-reactive protein (CRP) decreasing from 119 mg/L at admission to 85 mg/L on day 3 and 4.32 mg/L at discharge (day 6). Twenty-four hours after discharge, corresponding to seven days after IVIG administration, he was readmitted with a severe diffuse headache, three episodes of vomiting, marked fatigue, and a fever of 38.3°C. The chronological sequence of the patient's clinical course is summarized in Table 1.

**Table 1:** Timeline of the patient's clinical course from the diagnosis of Kawasaki disease to complete recovery. CRP: C-reactive protein; Hb: hemoglobin; ESR: erythrocyte sedimentation rate; IVIG: intravenous immunoglobulin

Time	Clinical course	Key investigations	Management	Outcome
Day 0	Persistent fever, bilateral non-purulent conjunctivitis, diffuse rash, pharyngitis, and abdominal pain	Elevated inflammatory markers (CRP 119 mg/L); echocardiography showed no coronary artery abnormalities	Hospital admission	Diagnosis of Kawasaki disease
Day 1	Stable clinical condition	Baseline laboratory evaluation (Hb 13.7 g/dL, platelets 217 × 10⁹/L, ferritin 175 ng/mL, triglycerides 0.55 g/L)	IVIG (2 g/kg over 10 hours), aspirin (50 mg/kg/day), and oral prednisone (1 mg/kg/day)	Good clinical response
Days 2–6	Progressive resolution of fever and mucocutaneous manifestations	CRP decreased to 85 mg/L on day 3 and 4.32 mg/L at discharge (day 6)	Continuation of treatment	Discharged home
Day 7	Readmission with severe headache, vomiting, fever (38.3°C), and mild neck stiffness	Leukocytosis (30,100/mm³), neutrophilia (21,500/mm³), thrombocytosis (461 × 10⁹/L), CRP 2.51 mg/L	Hospitalization and supportive care	Suspected aseptic meningitis
Day 7	Persistent meningeal symptoms	CSF: clear, 248 cells/mm³ (80% neutrophils), glucose 0.63 g/L, protein 0.42 g/L; Gram stain and bacterial cultures negative	Lumbar puncture and clinical monitoring	Infectious meningitis excluded
Days 8–9	Resolution of headache and vomiting	Normal complete blood count; CRP 2.2 mg/L; ESR 66 mm/h (first hour) and 90 mm/h (second hour)	Supportive care and observation	Complete recovery

On examination, the patient was conscious, alert, and hemodynamically stable. He had mild neck stiffness with no photophobia or focal neurological deficits. The remainder of the physical examination was normal.

Laboratory investigations revealed leukocytosis (30,100/mm³) with neutrophil predominance (21,500/mm³), thrombocytosis (461,000/mm³), and a CRP level of 2.51 mg/L. Serial laboratory findings throughout the clinical course are presented in Table 2.

**Table 2:** Relevant laboratory findings during the clinical course. ALT: alanine aminotransferase; AST: aspartate aminotransferase; CRP: C-reactive protein; ESR: erythrocyte sedimentation rate

Laboratory test	Day 0–1	Day 3	Day 6	Day 7	Days 8–9	Unit	Reference range
White blood cell count	29,060	20,240	31,470	30,100	14,360	/mm³	4,000–10,000
Neutrophil count	24,930	14,450	16,760	21,500	7,910	/mm³	1,500–7,000
Hemoglobin	13.7	10.9	13.4	14.5	14.2	g/dL	12.4-16.4
Platelet count	217,000	192,000	418,000	461,000	358,000	/mm³	150,000–400,000
CRP	119	85	4.32	2.51	2.2	mg/L	0–5
ESR	—	—	44	—	66	mm/h	0–15 mm in the 1st hour
Ferritin	175	269.96	—	—	—	ng/mL	22–275
Triglycerides	0.55	—	—	—	—	g/L	< 1.02
Urea	0.20	0.26	0.39	0.31	—	g/L	0.10–0.30
Creatinine	7.36	5.69	6.56	6.47	—	mg/L	5-10
AST	67	—	32	—	—	U/L	5–34
ALT	61	—	45	—	—	U/L	0–55

Lumbar puncture for suspicion of meningitis was performed. The clear CSF was characterized by pleocytosis (248 cells/mm³) with neutrophil predominance (80%), a glucose concentration of 0.63 g/L, and a protein concentration of 0.42 g/L. Gram stain and bacterial cultures were negative. The CSF findings are summarized in Table 3.

**Table 3:** Cerebrospinal fluid (CSF) findings at readmission.

CSF parameter	Patient value	Unit	Reference range
Macroscopic examination			
Appearance	Clear	—	—
Cytological examination			
Leukocyte count	248	cells/mm³	0–5
Red blood cell count	24	cells/mm³	0–5
Neutrophils	80	%	—
Lymphocytes	20	%	—
Biochemical examination			
Glucose	0.63	g/L	0.60–0.80
Protein	0.42	g/L	0.15–0.45
Chloride	122	mmol/L	110–130
Microbiological examination			
Gram stain	Negative	—	—
Bacterial culture	Negative	—	—

Additional CSF virological studies were not performed, as the clinical presentation, microbiological findings, and favorable evolution strongly supported the diagnosis of IVIG-induced aseptic meningitis.

Supportive care, including hydration, analgesics, and close clinical monitoring, was provided. His symptoms resolved rapidly, with complete resolution of headache and vomiting. At discharge, laboratory evaluation showed improvement in the white blood cell count to 14,360/mm³ and neutrophil count to 7,910/mm³, with a hemoglobin level of 14.2 g/dL, a platelet count of 358,000/mm³, and a CRP level of 2.2 mg/L. The patient was discharged in good clinical condition.

The diagnosis of IVIG-induced aseptic meningitis was supported by the close temporal relationship between IVIG administration and the onset of neurological symptoms, the characteristic CSF findings, negative microbiological investigations, and the rapid clinical improvement of the patient with supportive care.

At the one-week follow-up, the patient was clinically well and asymptomatic with no recurrence of neurological symptoms. Laboratory workup revealed hemoglobin of 13.4 g/dL, platelet count of 288,000/mm³, and CRP of 2 mg/L. At three-month follow-up, he was asymptomatic with an unremarkable clinical examination and normal echocardiogram. Antiplatelet-dose aspirin was discontinued after three months of treatment, and further clinical follow-up was planned.

## Discussion

Aseptic meningitis is an uncommon neurological complication of IVIG therapy with a reported incidence of 0.01%-1% [6,7]. However, its actual frequency is likely underestimated due to under-recognition and underreporting. Although IVIG is a safe and effective treatment for KD and other immune-mediated disorders, clinicians should be aware of this rare adverse event [2,3].

Symptoms generally occur within 24 to 72 hours after IVIG infusion, although delayed presentations up to seven days have been reported [6,7]. Meningeal symptoms in our patient appeared seven days after the administration of IVIG, which supports the fact that this diagnosis should still be considered in the differential when presentation is delayed.

The exact pathophysiologic mechanism is unknown. Proposed hypotheses have included delayed hypersensitivity reactions, cytokine-mediated inflammation, direct meningeal irritation by immunoglobulins, and neutrophil activation, but none has been definitively proven [3,4]. Several risk factors have been suggested, including high-dose IVIG (2 g/kg), rapid infusion rate, history of migraine, and underlying inflammatory disease [3,7]. However, aseptic meningitis can also be seen in patients without known predisposing factors, as in our case.

The clinical presentation resembles that of bacterial meningitis closely, with severe headache, fever, vomiting, photophobia, and neck stiffness, making the diagnosis particularly difficult [6]. Analysis of CSF usually shows pleocytosis with predominance of neutrophils, a mild increase in protein, normal glucose levels, and negative microbiological investigations [5,6]. These characteristic features were consistent with our patient’s clinical and laboratory findings. The most important differential diagnosis is bacterial meningitis, which is important to exclude because it is life-threatening. Table 4 summarizes the main clinical and laboratory features of both conditions.

**Table 4:** Differential diagnosis between IVIG-induced aseptic meningitis and bacterial meningitis. IVIG: intravenous immunoglobulin; CSF: cerebrospinal fluid; WBC: white blood cell

Feature	IVIG-induced aseptic meningitis	Bacterial meningitis
Onset	Within 1–7 days after IVIG infusion	No relation to IVIG
Fever	Mild to moderate	Usually high
Headache/Neck stiffness	Present	Present
CSF appearance	Usually clear	May be clear or turbid
CSF WBC	Pleocytosis, usually neutrophilic	Marked neutrophilic pleocytosis
CSF glucose	Normal	Low
CSF protein	Normal or mildly elevated	Markedly elevated
Gram stain/Culture	Negative	Often positive, but may be negative after antibiotic exposure
Treatment	Supportive care	Intravenous antibiotics
Outcome	Complete recovery	Variable; complications possible

Interestingly, our patient developed symptoms on day 7 of IVIG administration, which is relatively delayed compared to 24-72 hours in most pediatric cases [6,7]. Given the overlap between IVIG-induced aseptic meningitis and bacterial meningitis, a systematic diagnostic approach, including CSF analysis and exclusion of infectious causes, is essential for appropriate supportive management.

Management is mainly supportive, with exclusion of infectious meningitis and close clinical monitoring. Empirical antibiotic therapy is often started before microbiological results are available and should be stopped if bacterial infection has been ruled out [5,7]. Symptoms usually resolve within a few days without neurological sequelae, and the prognosis in most cases is good [7,8].

Our case underlines the importance of awareness of the rare but benign complication of IVIG therapy, aseptic meningitis, in children with KD. Early identification of this entity may avoid unnecessary workup, prolonged antibiotic use, and prolonged hospital stay while ensuring proper patient management [7,8].

From a clinical perspective, this case highlights several important lessons. Firstly, aseptic meningitis due to IVIG should be considered in the differential diagnosis of patients presenting with headache, fever, vomiting, and meningeal signs within days of high-dose IVIG administration even if the symptoms are delayed [6]. In contrast to most reported cases in which symptoms develop within 24 to 72 hours after IVIG infusion, our patient developed aseptic meningitis seven days after therapy, highlighting that this diagnosis should not be ruled out based on delayed presentation alone [5,6]. Secondly, in every case, diagnosis must exclude bacterial meningitis by appropriate investigation of CSF and microbiology [5,6]. Finally, the combination of marked neutrophilic pleocytosis with only mildly elevated inflammatory markers exemplifies the diagnostic difficulty of this condition [6]. Recognition of this self-limiting condition is important to prevent unnecessary interventions [7,8].

## Conclusions

IVIG-induced aseptic meningitis is a rare but important neurological complication of high-dose IVIG therapy in patients with KD. The diagnosis is based on the temporal relationship with IVIG administration, characteristic CSF findings, and the exclusion of infectious meningitis. Although symptoms typically occur within 24-72 hours after IVIG infusion, our case highlights that delayed presentations may also occur and should not exclude the diagnosis. Early recognition of this benign and self-limiting condition is essential to ensure appropriate management and avoid prolonged hospitalization. At the same time, appropriate supportive care remains essential, while awareness of its favorable prognosis may help clinicians reassure patients and their families.
